# Supplementary material for: Content-rich biological network constructed by mining PubMed abstracts
Source: BMC Bioinformatics. 2004 Oct 8;5:147. doi: 10.1186/1471-2105-5-147 (PMC528731; doi:10.1186/1471-2105-5-147)
Supplement: Additional File 2 — The original results of the above study (non-essential files are deleted to keep the file size under the limit set by BMC bioinformatics). [file 1471-2105-5-147-S2.bz2 › chilibotAdditionalFile2/dip50/274ID1922387E1094/html/MAPK9_JUN.html]

 


 **MAPK9** and **JUN** 
  
Found 316 abstracts in PubMed, retrieved 50.  
 

 What does Google say? 
 PDF only 
| .edu only 

---

**Interactive relationship** (e.g. stimulation, inhibition, etc)

**Stimulatory relationship**- JNK2  [ **MAPK9** ]  transcript inhibition abrogated shear induced COX 2, EP2 and EP3a1 mRNA upregulation, as well as c  **jun**  phosphorylation.  Ref: 12743126 J Biol Chem< MedlineTA>J Biol Chem,
**Neutral relationship**- Hypoxia led to significant activation of MAP kinase and  **Jun**  kinase  [ **MAPK9** ]  pathways, as shown by phosphorylated extracellular signal regulated kinase and  **Jun**  kinase  [ **MAPK9** ]  isotypes in male cells but this effect was modest in female cells.  Ref: 12374461 Endocrine, 2002

**Non-interactive relationship** (e.g. studied together, co-existance, homology, etc.)

- Increased expression of c  **jun**  N terminal kinase 2  [ **MAPK9** ] , integrin alpha E, MMP 8, MMP 9 was detected in both fractions of most patients.  Ref: 12152998 Leuk Lymphoma, 2002
- Exposure of T C 28a2 chondrocytic cells to fluid shear results in co regulation of c  **jun**  N terminal kinase2  [ **MAPK9** ]  JNK2  [ **MAPK9** ] , c  **jun**  and COX 2 as well as concomitant downstream expression of prostaglandin receptors EP2 and EP3a1.  Ref: 12743126 J Biol Chem< MedlineTA>J Biol Chem, 2002
- However, vanadate or acetyl furanonaphthoquinone induced the 40kDa Mcl 1 and greater  **Jun**  kinase  [ **MAPK9** ]  JNK phosphorylation without apoptosis associated PARP fragmentation in MCF 7 cells.  Ref: 12359245 Biochem Biophys Res Commun, 2002
- Thus, we suspected that either  **Jun**  N terminal kinase 2 JNK 2  [ **MAPK9** ]  or Src family proteins were involved in HLA DR mediated signaling.  Ref: 12842758 Cytokine, 2003
- **Jun**  kinase  [ **MAPK9** ]  modulates tumor necrosis factor dependent apoptosis in liver cells.  Ref: 12143039 Hepatology, 2002
- The reduction appeared to be due to relocalization of TNFRI from the cell surface and was reflected in the elimination of TNF alpha induced  **Jun**  kinase  [ **MAPK9** ]  activity.  Ref: 12768019 J Virol, 2003
- Tumor necrosis factor TNF triggers distinct pathways in liver cells through TNF receptor 1 TNF R1 via adapter molecules, including the intracellular cascades leading to apoptosis, nuclear factor kappa B NF kappa B, and  **Jun**  kinase  [ **MAPK9** ]  JNK activation.  Ref: 12143039 Hepatology, 2002
- Furthermore, ncp BVDV infection did not result in the activation of stress kinases JNK1 and JNK2  [ **MAPK9** ]  or the phosphorylation of transcription factors ATF 2 and c  **Jun** .  Ref: 12186882 J Virol, 2002
- Here we demonstrate in neonatal rat cardiomyocytes that 1 TNF alpha induces phosphorylation of AKT, activation of NF kappaB, and the phosphorylation of  **JUN**  kinase  [ **MAPK9** ] .  Ref: 12409315 FASEB J, 2002
- Hypertonicity induced by NaCl, but not by urea or mannitol, up regulates expression of the gamma subunit of Na K ATPase in cells of the murine inner medullary collecting duct line IMCD3 by activation of the  **Jun**  kinase  [ **MAPK9** ]  2 JNK2  [ **MAPK9** ]  pathways.  Ref: 12746499 Proc Natl Acad Sci U S A< MedlineTA>Proc Natl Acad Sci U S A, 2002
- Induction of experimental autoimmune encephalomyelitis in the absence of c  **Jun**  N terminal kinase 2  [ **MAPK9** ] .  Ref: 12147621 Int Immunol, 2002
- Further studies with the inhibitor of  **Jun**  kinase  [ **MAPK9** ]  JNK and phosphorylation null mutant of Bcl xL reveal the augmentative role of JNK mediated Bcl xL phosphorylation in apoptosis of prostate cancer cells.  Ref: 12633850 FEBS Lett, 2003
- Shear induced cyclooxygenase 2 via a JNK2  [ **MAPK9** ]  c  **jun**  dependent pathway regulates prostaglandin receptor expression in chondrocytic cells.  Ref: 12743126 J Biol Chem< MedlineTA>J Biol Chem, 2003
- Like ligand activated CD40, LMP 1 activates NF kappaB and  **Jun**  kinase  [ **MAPK9** ]  signaling pathways via binding, as a constitutive oligomer, to tumor necrosis factor receptor associated factors TRAFs.  Ref: 12610149 J Virol, 2003
- By immunoblotting, these proteins were identified as c  **Jun**  N terminal kinase JNK 2  [ **MAPK9** ]  and p38 mitogen activated protein kinase MAPK delta, respectively, both of which are believed to be involved in apoptosis induction by CDDP.  Ref: 12824923 Cancer Sci< MedlineTA>Cancer Sci, 2003
- 5 dihydroxyvitamin D3 inhibits ultraviolet B induced apoptosis,  **Jun**  kinase  [ **MAPK9** ]  activation, and interleukin 6 production in primary human keratinocytes.  Ref: 12858333 J Cell BiochemJ Cell Biochem, 2003
- Furthermore, CD40 stimulation led to phosphorylation of c  **Jun**  on its activation domain, implicating CD40 mediated  **Jun**  kinase  [ **MAPK9** ]  activation in the transcriptional regulation of IL 6 production.  Ref: 12626566 J Immunol, 2003
- 3 Through activation of the  **Jun**  kinase  [ **MAPK9** ]  pathway AP 1  [ **JUN** ]  is induced, leading to cell cycle arrest in the G0 G1 phase.  Ref: 12790790 Endocr Relat Cancer, 2003
- 100 108, which interacts with SOS,  **jun**  and  **jun**  kinase  [ **MAPK9** ]  JNK.  Ref: 12206509 J Protein Chem, 2002
- While we found no evidence for alterations in the activity of p38, we could detect an increased activity of  **Jun**  kinase  [ **MAPK9** ]  in senescent fibroblasts.  Ref: 12470828 Exp Gerontol< MedlineTA>Exp Gerontol, 2002
- We have also found that IFNalpha induced apoptosis depends upon activation of the NH 2 terminal  **Jun**  kinase  [ **MAPK9** ]  1 Jnk 1 and p 38 mitogen activated protein kinase,.  Ref: 12700650 Cell Death Differ, 2003
- These same peptides also inhibited JNK2  [ **MAPK9** ]  mediated phosphorylation of c  **Jun**  and ATF2, suggesting that transcription factors, MKK4, and the JIP scaffold compete for docking to JNK.  Ref: 12788955 J Biol Chem< MedlineTA>J Biol Chem, 2003
- Mechanical strain also activated N terminal  **Jun**  kinase  [ **MAPK9** ]  JNK that, in contrast, did not return to baseline during 24 h of continuous strain.  Ref: 12162499 J Bone Miner Res, 2002
- Tumor necrosis factor TNF signaling through the TNF receptors involves the recruitment of key signaling factors, leading to the activation of both the transcription factor NF kappaB and the stress activated  **Jun**  kinase  [ **MAPK9** ]  JNK.  Ref: 12417721 Mol Cell Biol, 2002
- The accumulation of ROS in senescent cells may be related to the constitutive activation of  **Jun**  kinase  [ **MAPK9** ] .  Ref: 12470828 Exp Gerontol< MedlineTA>Exp Gerontol, 2002
- The expression of c  **Jun**  N terminal protein kinase JNK1, but not JNK2  [ **MAPK9** ] , was increased 2.5 fold in malignant as compared to normal breast tissue.  Ref: 12618338 Cancer Lett, 2003
- Immunoblot analysis showed that either treatment attenuated constitutive phosphorylation of extracellular signal regulated kinase ERK 1 and ERK2 but not of p38 mitogen activated protein kinase and c  **Jun**  N terminal kinase JNK 1 and JNK2  [ **MAPK9** ] .  Ref: 12695541 Mol Pharmacol, 2003
- Further studies on these pathways showed that PS 341 decreases the levels of several antiapoptotic proteins and triggers a dual apoptotic pathway of mitochondrial cytochrome c release and caspase 9 activation, as well as activation of  **Jun**  kinase  [ **MAPK9** ]  and a Fas caspase 8 dependent apoptotic pathway which is inhibited by a dominant negative decoy Fas construct.  Ref: 12391322 Proc Natl Acad Sci U S A, 2002
- The p65 RelA subunit of NF kappaB suppresses the sustained, antiapoptotic activity of  **Jun**  kinase  [ **MAPK9** ]  induced by tumor necrosis factor.  Ref: 12417721 Mol Cell Biol, 2002
- The activation of one of these, the  **Jun**  kinase  [ **MAPK9** ]  pathway, has been implicated in apoptotic responses to DNA damage, cell stress and cytotoxic drugs.  Ref: 12860462 Drug Resist Updat, 2003
- Hence, a biochemical pathway exists wherein fluid shear activates COX 2, via a JNK2  [ **MAPK9** ]  c  **jun**  dependent pathway, which in turn elicits downstream EP2 and EP3a1 mRNA synthesis.  Ref: 12743126 J Biol Chem< MedlineTA>J Biol Chem, 2003
- A role for c  **Jun**  N terminal kinase 1 JNK1, but not JNK2  [ **MAPK9** ] , in the beta amyloid mediated stabilization of protein p53 and induction of the apoptotic cascade in cultured cortical neurons.  Ref: 12534344 Biochem J, 2003
- Relationship of Mcl 1 isoforms, ratio p21WAF1 cyclin A, and  **Jun**  kinase  [ **MAPK9** ]  phosphorylation to apoptosis in human breast carcinomas.  Ref: 12359245 Biochem Biophys Res Commun, 2002
- Positive immunostaining for JNK1, JNK2  [ **MAPK9** ] , p JNK, c  **Jun** , pc  **Jun** , c Fos, and alpha NAC was observed in 8 9, and 97.5% of the samples, respectively.  Ref: 12689679 Bone, 2003
- Functional knock out experiments using an antisense c  **jun**  oligonucleotide revealed the abolition of shear induced COX 2, EP2 and EP3a1, but not JNK2  [ **MAPK9** ] , transcripts.  Ref: 12743126 J Biol Chem< MedlineTA>J Biol Chem, 2003
- Studies on the potential signaling mechanisms demonstrated that in cells expressing inhibitor resistant Bcr Abl mutants, PP1 and CGP76030 inhibited the activity of Src family tyrosine kinases and Akt but not signal transducer and activator of transcription 5 STAT5 and  **JUN**  kinase  [ **MAPK9** ]  Jnk.  Ref: 12393636 Blood, 2003
- Role of  **Jun**  and  **Jun**  kinase  [ **MAPK9** ]  in resistance of cancer cells to therapy.  Ref: 12860462 Drug Resist Updat, 2003
